# β_2_-Glycoprotein I Inhibits Vascular Endothelial Growth Factor-Induced Angiogenesis by Suppressing the Phosphorylation of Extracellular Signal-Regulated Kinase 1/2, Akt, and Endothelial Nitric Oxide Synthase

**DOI:** 10.1371/journal.pone.0161950

**Published:** 2016-08-31

**Authors:** Wen-Chin Chiu, Tzeon-Jye Chiou, Meng-Ju Chung, An-Na Chiang

**Affiliations:** 1 Institute of Biochemistry and Molecular Biology, National Yang-Ming University, Taipei, Taiwan; 2 Division of Thoracic Surgery, Department of Surgery, Kaohsiung Medical University Hospital, Kaohsiung Medical University, Kaohsiung, Taiwan; 3 Division of Transfusion Medicine, Department of Medicine, Taipei Veterans General Hospital and National Yang-Ming University School of Medicine, Taipei, Taiwan; Medical University Innsbruck, AUSTRIA

## Abstract

Angiogenesis is the process of new blood vessel formation, and it plays a key role in various physiological and pathological conditions. The β_2_-glycoprotein I (β_2_-GPI) is a plasma glycoprotein with multiple biological functions, some of which remain to be elucidated. This study aimed to identify the contribution of _2_-GPI on the angiogenesis induced by vascular endothelial growth factor (VEGF), a pro-angiogenic factor that may regulate endothelial remodeling, and its underlying mechanism. Our results revealed that β_2_-GPI dose-dependently decreased the VEGF-induced increase in endothelial cell proliferation, using the 3-(4,5-dimethylthiazol-2-yl)-2,5-diphenyltetrazolium bromide (MTT) and the bromodeoxyuridine (BrdU) incorporation assays. Furthermore, incubation with both β_2_-GPI and deglycosylated β_2_-GPI inhibited the VEGF-induced tube formation. Our results suggest that the carbohydrate residues of β_2_-GPI do not participate in the function of anti-angiogenesis. Using *in vivo* Matrigel plug and angioreactor assays, we show that β_2_-GPI remarkably inhibited the VEGF-induced angiogenesis at a physiological concentration. Moreover, β_2_-GPI inhibited the VEGF-induced phosphorylation of extracellular signal-regulated kinase 1/2 (ERK1/2), Akt, and endothelial nitric oxide synthase (eNOS). In summary, our *in vitro* and *in vivo* data reveal for the first time that β_2_-GPI inhibits the VEGF-induced angiogenesis and highlights the potential for β_2_-GPI in anti-angiogenic therapy.

## Introduction

β_2_-glycoprotein I (β_2_-GPI) is a 50 kDa plasma glycoprotein possessing 326 amino acids with 5 homologous domains and four N-glycosylation sites [1–3]. The functions of β_2_-GPI are involved in a variety of physiological processes including triglyceride metabolism, vascular homeostasis, and blood coagulation [4,5]. Our previous studies demonstrated that β_2_-GPI induces endothelial nitric oxide synthase (eNOS) activation and nitric oxide (NO) production through the NF-κB signaling pathway, thereby modulating vascular cell migration [6]. The endothelium serves as an interface between the circulating blood system and vascular homeostasis. Several studies have shown that _2_-GPI could bind to endothelial cells through candidate receptors [7,8], although the underlying mechanism activated by _2_-GPI in endothelial cells remains unknown.

Vascular endothelial growth factor (VEGF) signaling has an important role as a pro-angiogenic factor, permitting physiological revascularization. Therefore, drug or biological components targeting the VEGF signaling pathway have been extensively used as potential anti-angiogenic agents [9,10]. Recently, we found that _2_-GPI has the ability to inhibit the VEGF-induced cell growth and migration in human aortic endothelial cells (HAECs) [11]. Alterations in endothelial cell migration and proliferation are associated with diverse vascular pathologies such as angiogenesis, restenosis in grafted or injured vessels, and atherogenesis [12,13].

Angiogenesis plays a major role in the pathogenesis of several diseases such as rheumatoid arthritis [14], cerebral ischemia [15], tumor growth and metastasis [16], and wounded skin [17]. The main member of the VEGF family, VEGF-A (referred to as VEGF hereafter), has been shown to activate signaling enzymes including mitogen-activated protein kinase (MAPK), Akt, protein kinase C (PKC), and eNOS primarily through its receptor, VEGFR2 [18–20]. Recent studies have shown that activation of extracellular signal-regulated kinase (ERK)1/2 and Akt pathways is involved in the upregulation of VEGF and intervention of angiogenesis [21,22]. However, the molecular mechanisms by which β_2_-GPI regulates the VEGF-induced angiogenesis within vascular endothelial cells still remain unclear. Therefore, we aimed to determine the effect of β_2_-GPI on the VEGF-induced angiogenesis in HAECs; also, we investigated whether the phosphorylation of ERK1/2, Akt, and eNOS was regulated by β_2_-GPI. This study could provide new ideas for therapeutic strategies that ameliorate the vascular pathology observed in neovascularization and endothelial remodeling.

## Materials and Methods

### Reagents and antibodies

VEGF-A was purchased from Sigma-Aldrich (St. Louis, MO, USA). Rabbit anti-β_2_-GPI antibody was prepared as described previously [23]. The growth factor-reduced matrigel and the anti-eNOS antibody were purchased from BD Biosciences (Bedford, MA, USA). Antibodies against phospho-ERK1/2, phospho-Akt, phospho-eNOS, and ERK1/2 were purchased from Cell Signaling Technology (Beverly, MA, USA). The antibody against Akt was purchased from Santa Cruz Biotechnology (Santa Cruz, CA, USA).

### Cell culture

Human aortic endothelial cells (HAECs) were purchased from Cascade Biologics (Portland, OR, USA) and were cultured at 37°C in Medium 200 (Cascade Biologics) supplemented with low serum growth supplement (LSGS, Cascade Biologics) containing 2% fetal bovine serum (FBS), 1 μg/ml hydrocortisone, 10 ng/ml human epidermal growth factor, 3 ng/ml basic fibroblast growth factor, 10 μg/ml heparin, and 1% antibiotic mixture according to the manufacturer’s instructions.

### Purification and deglycosylation of β_2_-GPI

β_2_-GPI was purified from human plasma by methods that have been previously used [6]. Briefly, β_2_-GPI was isolated and purified by a 3% perchloric acid precipitation and a Heparin-Sepharose affinity chromatography (HiTrap Heparin, GE healthcare Bio-Sciences, Uppsala, Sweden). The purity of the β_2_-GPI was determined through 10% SDS-PAGE and Western blot analysis. The purified β_2_-GPI showed a single band in the SDS-PAGE, at approximately 50 kDa. For the deglycosylation assay, β_2_-GPI was denatured in 0.5% SDS and 40 mM DTT at 37°C for 10 min. After boiling the sample, peptide-N-glycosidase F (PNGase F, New England Biolabs, Ipswich, MA, USA) was added and incubated at 37°C for 72 h. The deglycosylated β_2_-GPI was detected by SDS–PAGE.

### Cell proliferation assay

HAECs were seeded in 96-well plates (2 × 10^4^ cells/well) and incubated in media with a LSGS containing 0.5% FBS for 24 h at 37°C. After treatment of β_2_-GPI or anti-β_2_-GPI antibody in the presence or absence of VEGF for 72 h, cells were incubated with 3-(4,5-dimethylthiazol-2-yl)-2,5-diphenyl tetrazolium bromide (MTT, 0.5 mg/ml, Sigma, St. Louis, MO, USA) for another 4 h. Then, medium was removed and the formazan crystals were dissolved in isopropanol. The amount of solubilized blue formazan was quantified by previously described methods [6]. Albumin (200 μg/ml) was used as a control protein. For the bromodeoxyuridine (BrdU) incorporation assay, cells were cultured on a 96-well plate and incubated with β_2_-GPI or anti-β_2_-GPI antibody in the presence of VEGF for 72 h. Cells were then labeled with BrdU and quantification was performed using a cell proliferation ELISA colorimetric kit (Roche Applied Science, Mannheim, Germany) according to the manufacturer’s instructions.

### In vitro angiogenic tube formation assay

The μ-slide (ibidi GmbH, Martinsried, Germany) coated with growth factor-reduced basement membrane extract (BME, Trevigen, Gaithersburg, MD, USA) was allowed to polymerize at 37°C for 30 min. Cells (7×10^3^ cells/well) were plated onto the μ-slide and treated with β_2_-GPI and VEGF for 14 h. After incubation, the morphology of cells was visualized, the degree of tube formation in each group was estimated by the presence of total length, and images were analyzed by the Metamorph tube formation module (Molecular Devices, San Diego, CA, USA).

### Animals

Forty-eight male C57BL/6 mice (6- to 8-weeks-old; Jackson Laboratories, Bar Harbor, ME, USA) were randomly allocated to one of the four groups (n = 6). Mice were housed with sterilized stainless steel cover and bedding, under 12 hour circadian cycle of artificial light, 22±2°C temperature, and 40–60% relative humidity. Food and drinking water were supplied *ad libitum*. All experiments involving mice were approved by the Institutional Animal Care and the Use Committee (IACUC) of National Yang-Ming University. The care of animals was conducted in accordance with the guidelines established by the U.S. National Institutes of Health Guide for the Care and Use of Laboratory Animals.

### Angiogenesis assay

For the *in vivo* Matrigel plug assay, male C57BL/6 mice were anesthetized by intraperitoneal (i.p.) injection of 15 μl of 2.5% avertin before the experiment. Following euthanasia, mice were injected subcutaneously with 500 μl Matrigel containing VEGF (20 ng/ml), heparin (50 U/ml), and either β_2_-GPI (200 μg/ml) or phosphate buffered saline (PBS) as a control. After 14 days, mice were euthanized by CO_2_ inhalation and Matrigel plugs were dissected out to quantify hemoglobin using the Drabkin’s reagent kit (Sigma, St. Louis, MO, USA) according to the manufacturer’s instructions. As a separate experiment of the *in vivo* angioreactor angiogenesis assay, we performed a procedure identical to the one described above, except for the Matrigel with or without VEGF and β_2_-GPI, which was put into sterilized angioreactors. Physical appearance, behavior and local clinical signs of the animals were daily observed throughout experiment. Mice were sacrificed by CO_2_ inhalation if they became clinically ill (weight loss more than 20% or hunching behavior). All mice were treated humanely throughout the experimental period.

### Western blot analysis

The effects of β_2_-GPI on the VEGF-induced expression of cellular signaling proteins were determined by Western blot analysis. HAECs were lysed in a buffer containing 1% Triton X-100, 50 mM HEPES, 6 mM EDTA, a phosphatase inhibitor (Sigma, St. Louis, MO, USA), and a complete protease inhibitor cocktail (Roche Applied Science, Mannheim, Germany). Whole cell extracts were collected by centrifugation at 12,000 × *g* for 20 min at 4°C. Protein concentration was determined using the Bradford assay (Bio-Rad, Hercules, CA), with BSA as a standard. Equal amount of proteins were subjected to 10% SDS-PAGE and transferred onto nitrocellulose membranes (Pall corporation, Pensacola, FL, USA) after gel electrophoresis. Immunoblots were blocked with 5% non-fat milk for 1 h and then incubated with primary antibodies for 16 h at 4°C. After washing, the transferred blots were incubated with horseradish peroxidase (HRP)-conjugated secondary antibodies (Sigma, St. Louis, MO, USA) for 1 h at 4°C. The bound IgG protein bands were visualized using an enhanced chemiluminescence detection kit system (ECL, PerkinElmer, Boston, MA, USA). The relative intensity of the protein bands was quantified by densitometry using the Image Quant software (Molecular Dynamics, Sunnyvale, CA, USA).

### Statistical analysis

The results are expressed as mean ± SEM of at least three independent experiments. A Student's *t*-test was used to evaluate statistically significant differences between two groups. Statistical analyses between three or more groups were performed using one-way ANOVA with Tukey's method as a *post hoc* test. A *p* < 0.05 was considered statistically significant.

## Results

### Both β_2_-GPI and deglycosylated β_2_-GPI inhibit the VEGF-induced cell proliferation, tube formation, and angiogenesis

**Fig 1:**
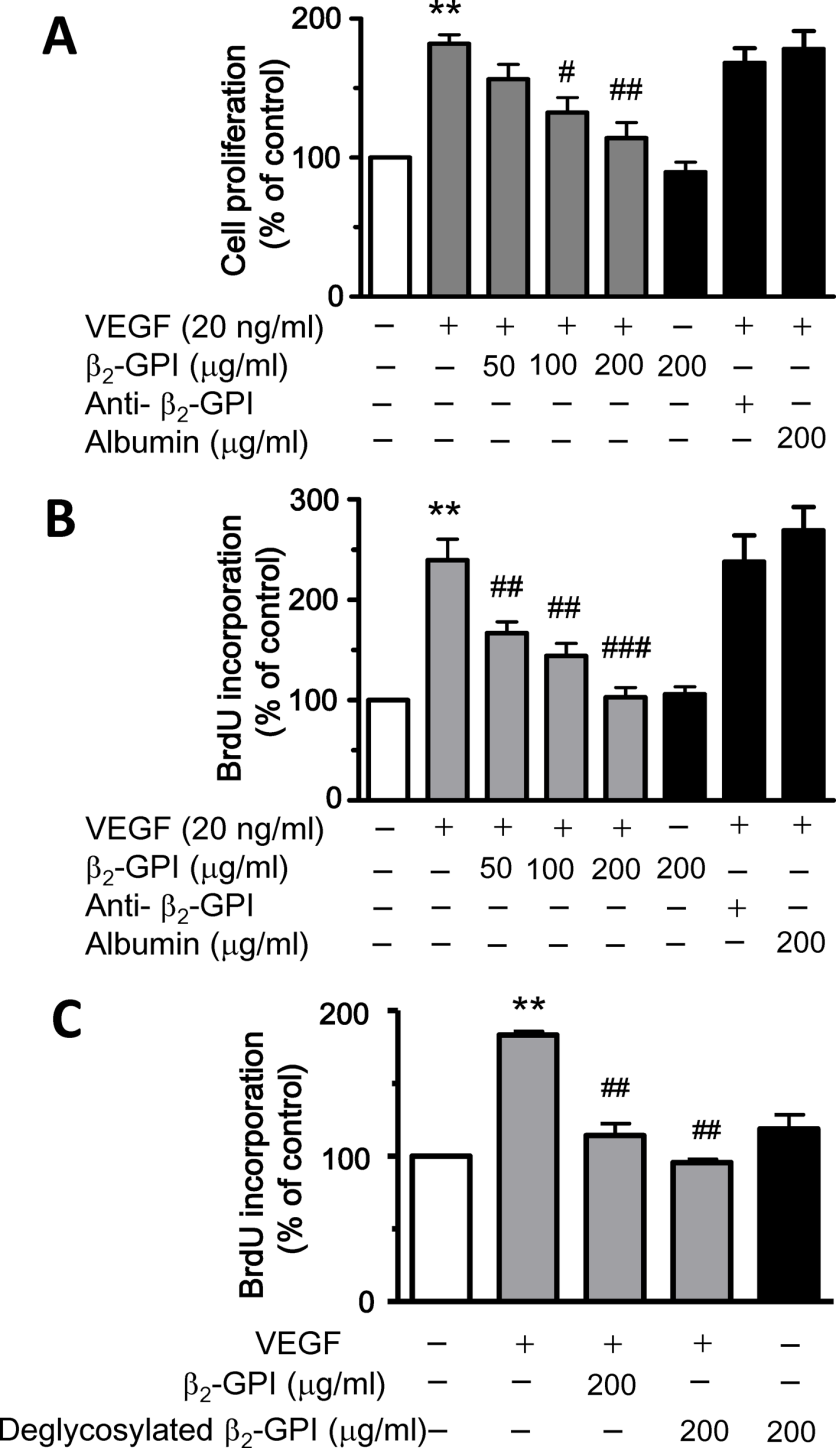
β_2_-GPI and deglycosylated β_2_-GPI inhibits the VEGF-induced proliferation in HAECs. (A) HAECs were treated with or without VEGF in combination with β_2_-GPI at indicated concentrations for 72 h and were incubated with 3-(4,5-dimethylthiazol-2-yl)-2,5-diphenyltetrazolium bromide (MTT) for another 4 h. Then the effect of β_2_-GPI on VEGF-induced cell proliferation was determined by MTT assay. Treatment of anti-β_2_-GPI antibody and albumin was performed to confirm the specific effect of β_2_-GPI on cell proliferation. (B) The effect of β_2_-GPI on VEGF-induced cell proliferation was also determined by BrdU incorporation assay in HAECs with or without VEGF. Cells were cultured on a 96-well plate and were incubated with β_2_-GPI in the presence of VEGF for 72 h, and then labeled with BrdU. Quantification was performed using a cell proliferation ELISA colorimetric kit. Treatment of anti-β_2_-GPI antibody and albumin was used as the comparison group. (C) The purified β_2_-GPI was denatured and the carbohydrate residues of β_2_-GPI were removed by peptide-N-glycosidase F. Then the effect of β_2_-GPI and deglycosylated β_2_-GPI at 200 μg/ml on cell proliferation was compared to the cells treated with VEGF by BrdU incorporation assay. Statistics were done using one-way ANOVA and data are expressed as a percentage normalized to the control group (set as 100%). Results are expressed as mean ± SEM of at least three independent experiments. **p < 0.01 versus control group; ^#^*p* < 0.05, ^##^*p* < 0.01, ^###^*p* < 0.001 versus VEGF treatment alone.

**Fig 2:**
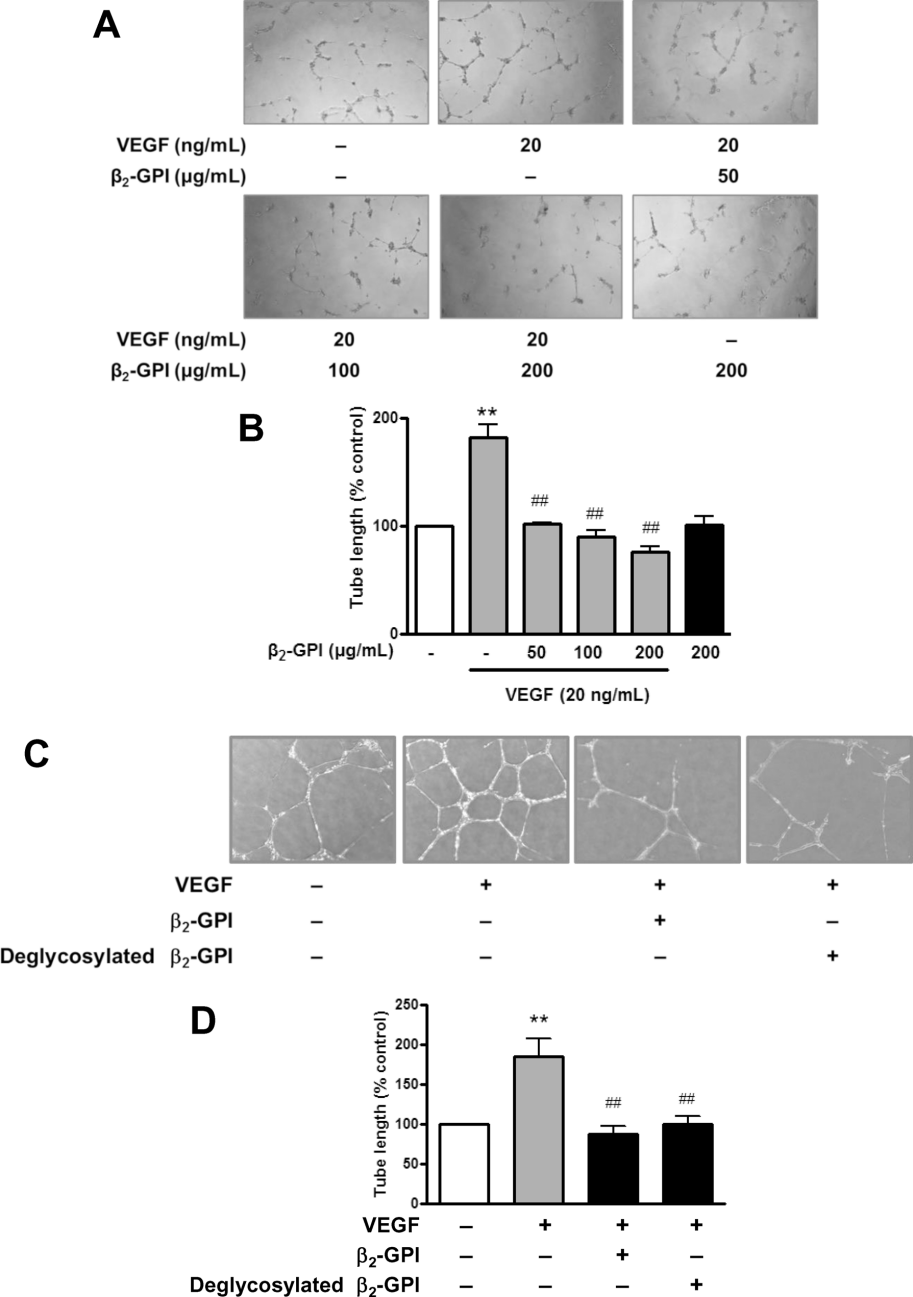
β_2_-GPI and deglycosylated β_2_-GPI inhibits the VEGF-induced tube formation in HAECs. (A) HAECs were seeded on the surface of a basement membrane extract and were treated with or without VEGF in combination with β_2_-GPI at indicated concentrations. The results are representative of those observed in four separate experiments (×40). (B) The degree of tube formation in HAECs was estimated by the Metamorph tube formation module. Bar graphs represent the quantitative analysis of tube formation. (C) Images were taken in HAECs treated with or without VEGF in combination with β_2_-GPI and deglycosylated β_2_-GPI. The results are representative of those observed in four separate experiments. (D) The effect of β_2_-GPI and deglycosylated β_2_-GPI on tube formation in HAECs was estimated by the Metamorph tube formation module. Bar graphs represent the quantitative analysis of tube formation expressed as mean ± SEM and representative of more than three independent experiments. **p < 0.01 versus control; ^##^*p* < 0.01 versus VEGF treatment alone.

Incubation with β_2_-GPI dose-dependently decreased the VEGF-induced proliferation of HAECs (Fig 1A and 1B). However, the suppressive effect of β_2_-GPI was not shown in cells without VEGF treatment. Treatment of an anti-β_2_-GPI antibody and albumin did not show the inhibitory effect on VEGF-induced proliferation. However, the VEGF-induced cell proliferation was inhibited by treatment with the deglycosylated β_2_-GPI (Fig 1C). An *in vitro* tube formation assay was used to evaluate the role of β_2_-GPI in the angiogenic activity in HAECs. Both β_2_-GPI (50, 100, 200 μg/ml) and deglycosylated β_2_-GPI (200 μg/ml) significantly inhibited the VEGF-induced tube formation (Fig 2).

**Fig 3:**
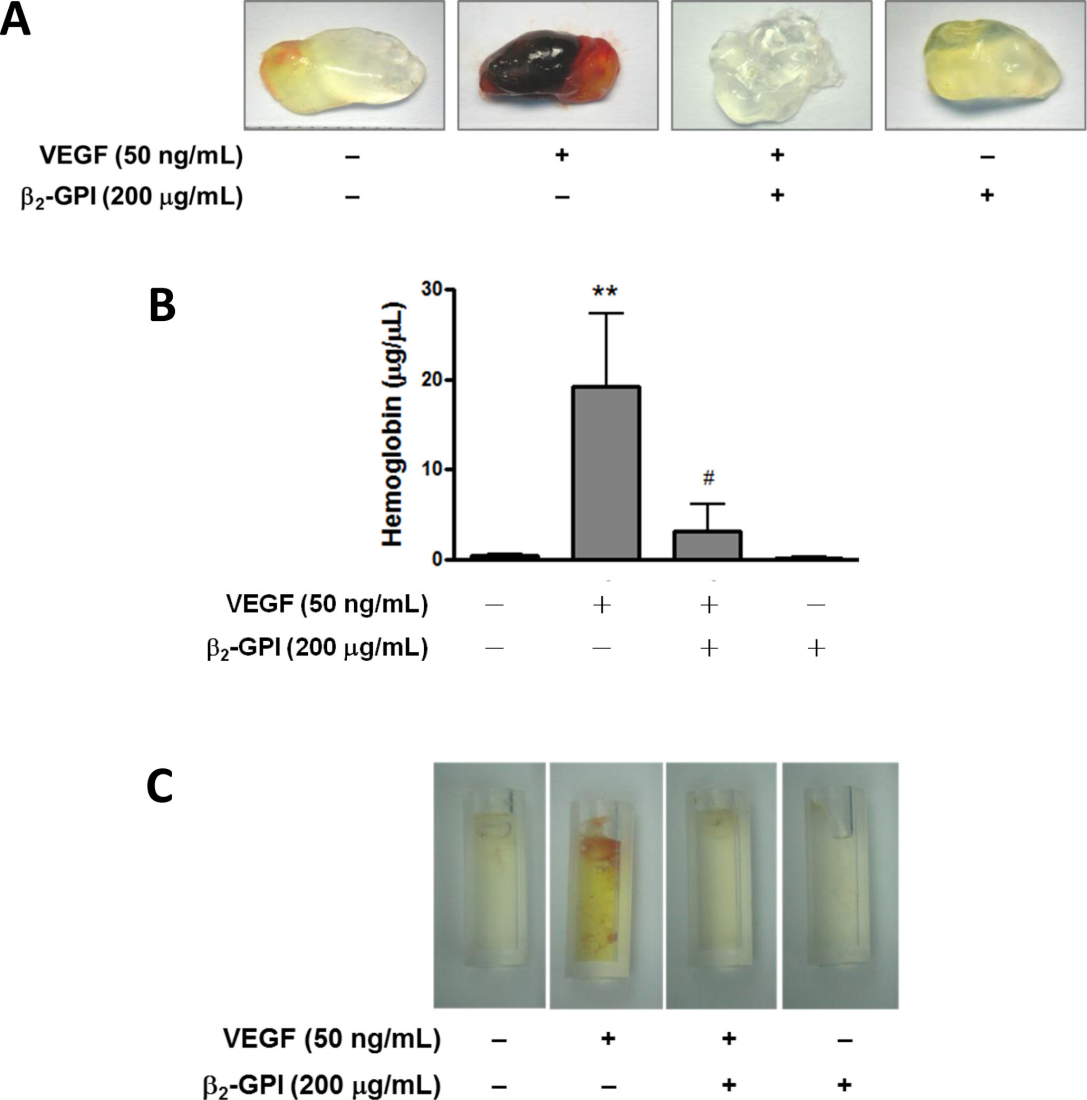
β_2_-GPI inhibits the VEGF-induced angiogenesis in mice. (A) C57BL/6 mice were injected subcutaneously with 0.5 ml Matrigel containing with or without VEGF in combination with β_2_-GPI at indicated concentrations (n = 6–8 per group). After 14 days, Matrigel plugs were removed and representative images were taken as shown. (B) Quantitative evaluation of angiogenesis in Matrigel plugs was determined by hemoglobin using the Drabkin’s reagent kit. Bar graphs represent the quantitative analysis of the hemoglobin content of plugs expressed as mean ± SEM. **p < 0.01 versus control; ^#^*p* < 0.05 versus VEGF treatment alone. (C) The effect of β_2_-GPI on the VEGF-induced angiogenesis was also detected using an angioreactor-based *in vivo* assay. Angioreactors with or without VEGF in combination with β2-GPI were implanted subcutaneously into the dorsal flank of C57BL/6 mice for 14 days, and vessels allowed to infiltrate. Two silicone tubes were implanted per mouse. Angioreactors are photographed using a Canon powershot G9 digital camera and the representative images were taken as shown.

The effect of β_2_-GPI on angiogenesis, *in vivo*, was also determined using a mouse model implanted with matrigel plugs. We observed that hemoglobin levels in the plugs containing β_2_-GPI and VEGF in mice was significantly lower when compared to plugs containing only VEGF (Fig 3A and 3B). As an alternative approach, we used angioreactors and demonstrated that β_2_-GPI had the same inhibitory effect on the VEGF-induced angiogenesis (Fig 3C), suggesting that β_2_-GPI plays an essential role in the inhibition of neovascularization. Taken together, these results provide evidence that both β_2_-GPI and deglycosylated β_2_-GPI inhibit the VEGF-induced cell growth and angiogenesis.

### Effects of β_2_-GPI on the VEGF-induced ERK1/2 and Akt expression in HAECs

**Fig 4:**
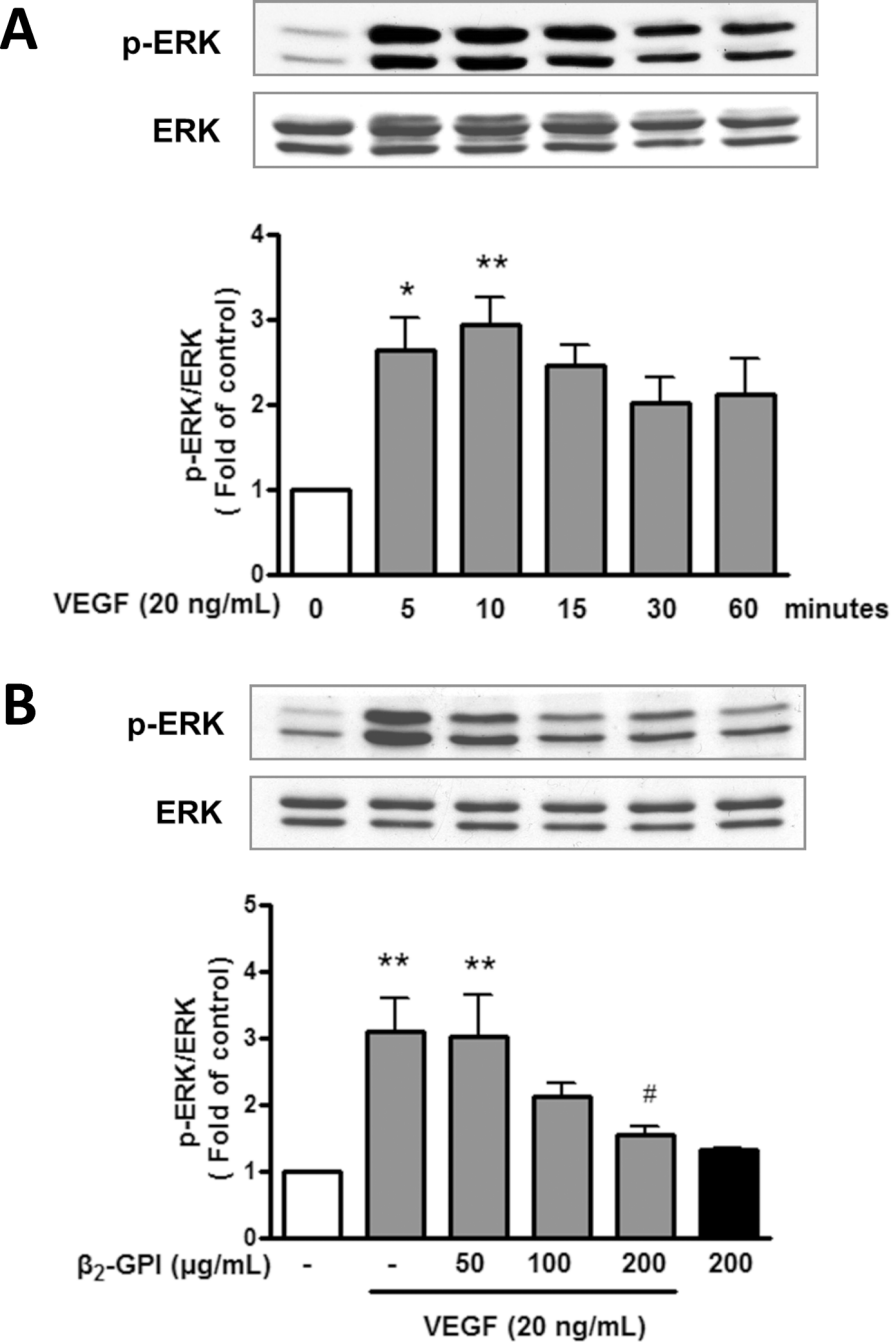
β_2_-GPI inhibits the VEGF-induced ERK1/2 phosphorylation in HAECs. (A) A time course of ERK1/2 phosphorylation in HAECs treated with VEGF was performed and monitored by Western blot analysis. The intensity of ERK1/2 phosphorylation band was normalized against total ERK1/2 expression and was calculated as an expression fold (relative to the control, which was set as 1). (B) The effect of β_2_-GPI on the VEGF-induced ERK1/2 phosphorylation was determined in HAECs treated with or without VEGF in combination with β_2_-GPI at indicated concentrations. Results are expressed as mean ± SEM and representative of more than three independent experiments. *p < 0.05; **p < 0.01 versus control; ^#^*p* < 0.05 versus VEGF treatment alone.

**Fig 5:**
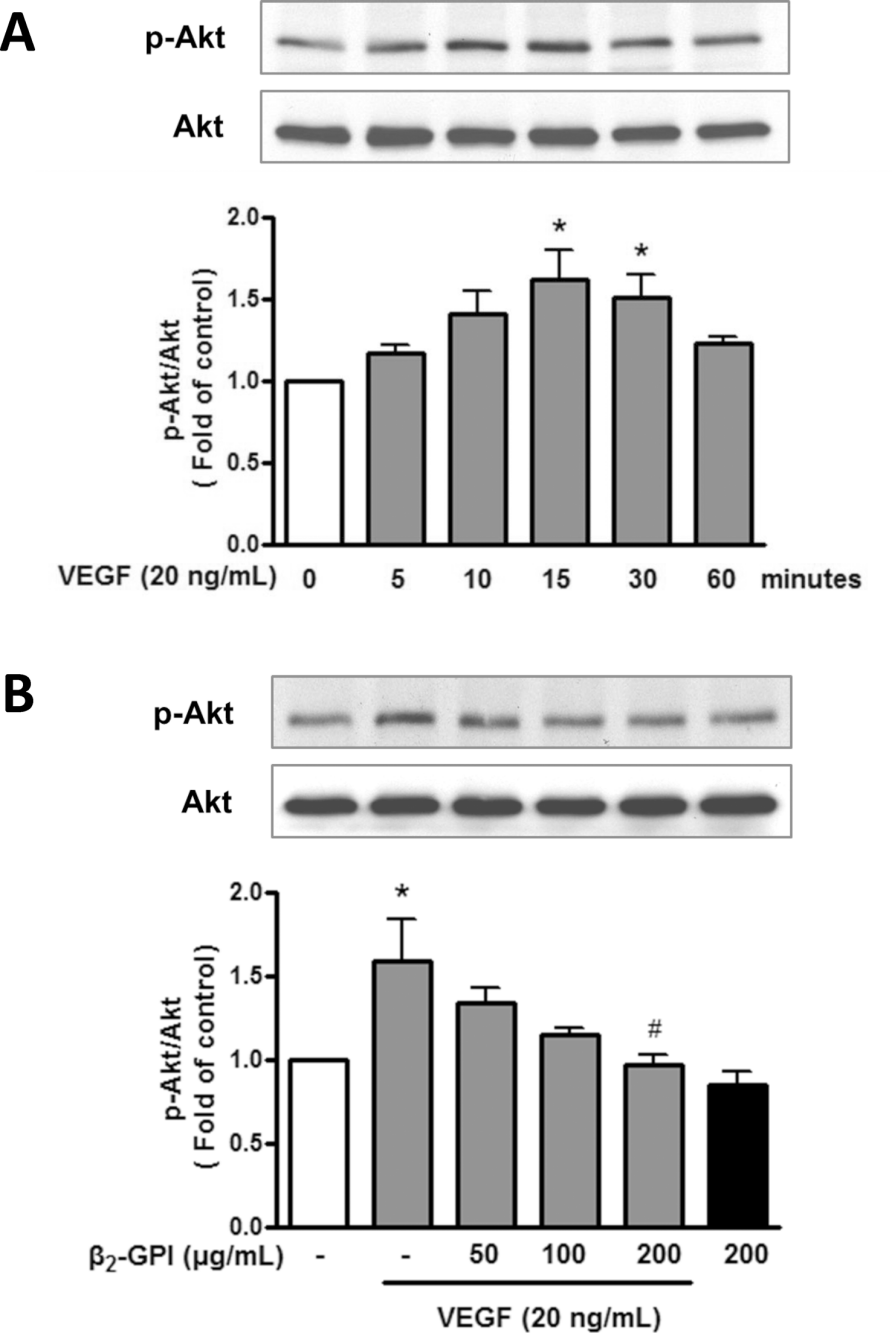
β_2_-GPI inhibits the VEGF-induced Akt phosphorylation in HAECs. (A) A time course of Akt phosphorylation in HAECs treated with VEGF was determined by Western blot analysis. The intensity of the Akt phosphorylation band was normalized against total Akt expression and was calculated as an expression fold (relative to the control, which was set as 1). (B) The effect of β_2_-GPI on VEGF-induced Akt phosphorylation was determined in HAECs treated with or without VEGF in combination with β_2_-GPI at indicated concentrations. Results are presented as mean ± SEM and representative of more than three independent experiments. *p < 0.05 versus control; ^#^*p* < 0.05 versus VEGF treatment alone.

To determine whether the inhibitory effect of β_2_-GPI on the VEGF-induced cell growth and angiogenesis is mediated through ERK1/2 and Akt pathways, we examined the expression levels of phosphorylated ERK1/2 and Akt in β_2_-GPI-treated cells. VEGF treatment induced the phosphorylation of ERK1/2 and Akt apparently after 10 min and 15 min, respectively (Figs 4A and 5A). Furthermore, β_2_-GPI treatment significantly attenuated the VEGF-induced ERK1/2 and Akt phosphorylation in a dose-dependent manner (Figs 4B and 5B). Expression levels of phospho-ERK1/2 and Akt were unaltered in β_2_-GPI-treated cells, when compared to the group without VEGF treatment. These findings clearly demonstrate that β_2_-GPI inhibits the VEGF-induced ERK1/2 and Akt phosphorylation in HAECs.

### β_2_-GPI decreases the VEGF-induced eNOS activation in HAECs

**Fig 6:**
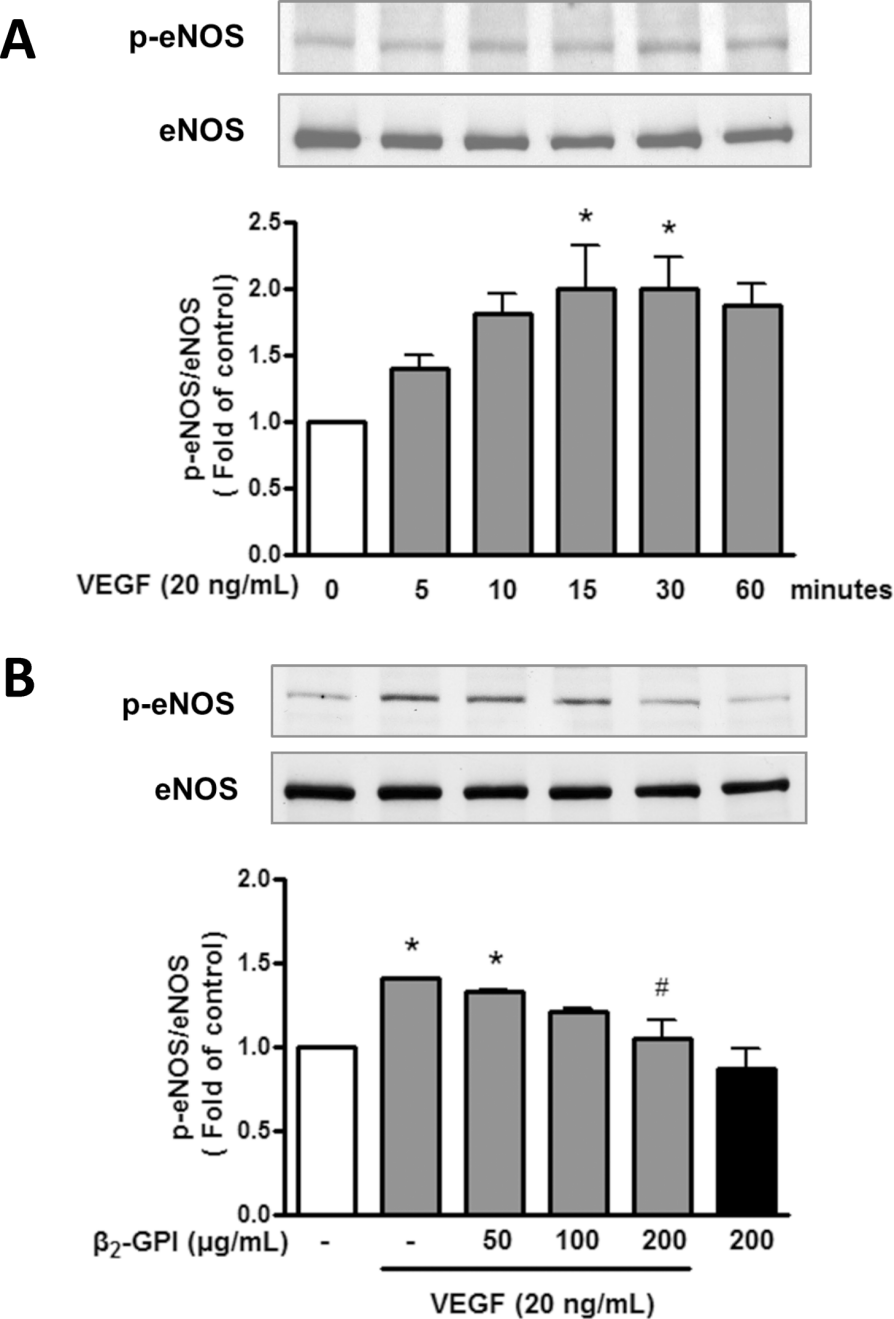
β_2_-GPI inhibits the VEGF-induced eNOS phosphorylation in HAECs. (A) A time course of eNOS phosphorylation in HAECs treated with VEGF was determined by Western blot analysis. The intensity of the eNOS phosphorylation band was normalized against total eNOS expression and was calculated as the fold of control (set as 1). (B) The effect of β_2_-GPI on VEGF-induced eNOS phosphorylation was determined in HAECs treated with or without VEGF in combination with β_2_-GPI at indicated concentrations. Results are expressed as mean ± SEM and representative of more than three independent experiments. *p < 0.05 versus control; ^#^*p* < 0.05 versus VEGF treatment alone.

We also examined whether β_2_-GPI could affect eNOS phosphrylation in HAECs. As shown in Fig 6A, the levels of phosphorylated eNOS at Ser^1177^ were highest after treatment with 20 ng/ml VEGF for 15–30 min. Furthermore, β_2_-GPI treatment dose-dependently decreased the stimulatory effect of VEGF on eNOS phosphorylation at 15 min (Fig 6B). In contrast, β_2_-GPI treatment alone had no effect on the phosphorylation of eNOS (when compared to the group without VEGF treatment). These results show that β_2_-GPI inhibits the VEGF-induced eNOS phosphorylation in HAECs.

## Discussion

Neovascularization is associated with diverse pathological processes such as atherosclerotic plaque rupture, ischemic retinopathies, and carcinogenesis [24–26]. Angiogenesis is a main process of neovascularization, therefore, management of angiogenesis is a high value therapeutic approach. Although we have reported that β_2_-GPI is able to inhibit endothelial migration and VEGF-induced cell growth [6,11], the effect of β_2_-GPI on angiogenesis of HAECs is still unknown. β_2_-GPI is a glycoprotein with a circulating concentration of approximately 200 μg/ml in human plasma [27]. We postulated that physiological concentrations of β_2_-GPI could alter endothelial cell function, which could prevent or ameliorate the vascular pathology observed in patients with angiogenesis or neovascularization. The results of this study support the idea that β_2_-GPI counteracts the adverse effects of the VEGF-induced angiogenesis in HAECs.

It has been shown that glycosylation affects the angiogenic activity of several proteins [28–30], although not all of the angiogenic regulation comes from the carbohydrate-residues of the proteins [31,32]. Several extracellular matrix proteins, such as endostatin, thrombospondin-1 (TSP-1), tumstatin, and their proteolytic fragments, have attracted considerable attention due to their anticancer effects, which are mainly attributed to the inhibition of tumor cell angiogenesis [33]. To investigate if the carbohydrate moieties of β_2_-GPI are involved in its anti-angiogenic activity, we determined the effect of a deglycosylated β_2_-GPI on angiogenic tube formation. We showed that the deglycosylated β_2_-GPI has the same inhibitory effect as β_2_-GPI in VEGF-treated HAECs. This suggests that the carbohydrate residues of β_2_-GPI are not involved in the VEGF-induced angiogenic activity, consistent with findings reported by Yu P et al., 2008 [34]. The _2_-GPI structure contains a distinct kringle domain at the C-terminal, which carries a lysine-rich sequence motif that binds negatively charged lipids or anionic lipid-containing target membranes [35,36]. Previous studies have shown that β_2_-GP1 binds to the surface of endothelial cells through TLR2 or annexin 2 [7,37]. Accumulated evidence shows that plasmin cleavage, which changes the intact form to the nicked form, results in a kringle domain alteration that dramatically switches the natural function of _2_-GPI in pathophysiological events [38].

β_2_-GPI behaves as a cell viability maintaining factor for endothelial cells [39]. Furthermore, Ioannou et al., (2010) reported that the free thiol form of β_2_-GPI has a protective effect against oxidative stress-induced endothelial cell death [40]. Recently, it has been demonstrated that increased microvessel formation occurs in the _2_-GPI-deficient mice [41]. Therefore, circulating levels of β_2_-GPI may play a role in vascular endothelial integrity. During fibrinolysis, fibrin-catalyzed cleavage of plasminogen produces clot-digesting plasmin and the antiangiogenic molecule, angiostatin [42]. Varying levels of a nicked β_2_-GPI, a protein form that has been proteolytically cleaved at Lys^317^/Thr^318^ residues, have been found in the plasma of leukemia patients [38]. Moreover, several studies have reported that this nicked β_2_-GPI is able to bind plasminogen and inhibits endothelial cell growth *in vitro*, and suppressed neovascularization and tumor growth *in vivo* [43–45]. These observations raise the possibility that the kringle domain may not be essential for the anti-angiogenic activity of β_2_-GPI. In the present study, we show that native β_2_-GPI suppresses the VEGF-induced endothelial cell proliferation and angiogenesis in HAECs. The antiangiogenic activity of endothelial cells provides a potential linkage to the inhibition of neovascularization *in vivo*. In this study, β_2_-GPI also shows a potent antiangiogenic activity *in vivo*, as demonstrated by matrigel plug and angioreactor assays.

Given the molecular basis underlying the inhibitory effects of β_2_-GPI in angiogenesis, we highlighted changes in its signaling pathway and attempted to predict its functional implications. VEGF is known to be one of the most potent angiogenic factors that promote cell migration, cell proliferation, tumor angiogenesis, and tumor cell growth [20, 46–49]. Although the VEGF signaling pathway in endothelial cells is not fully understood, molecules such as MAPK, phosphatidylinositol 3-kinase (PI3K)/Akt, Src, and eNOS/NO have been reported to be involved in the VEGF signaling pathway [18–20, 50–52]. Beecken et al., (2010) found that the nicked β_2_-GPI is able to inhibit endothelial cell growth through cyclin proteins and the MAPK signaling pathway [53]. Activation of ERK1/2 has also been associated with cell growth, migration, and morphogenesis induced by angiogenic factors [54,55]. On the other hand, activation of the PI3K/Akt signaling pathway has been associated with a variety of biological functions including cell growth, survival, vascular remodeling, and angiogenesis [55,56]. Moreover, endothelium-derived NO appears to play a role in angiogenesis, particularly in endothelial cell mobilization and tube formation [57–59]. Accumulating reports suggest that a decrease in ERK1/2, Akt, or eNOS activation is one possible approach in angiogenesis-dependent diseases [60–62]. In the present study, our data suggest that _2_-GPI inhibits the VEGF-induced angiogenesis by suppressing the phosphorylation of ERK1/2, Akt, and eNOS in HAECs.

In summary, our study provides evidence demonstrating that β_2_-GPI suppresses the VEGF-induced angiogenesis *in vitro* and *in vivo*. Furthermore, we shed light to the mechanisms by which β_2_-GPI affects its underlying signaling pathways; specifically, by suppressing the phosphorylation of ERK1/2, Akt, and eNOS. These results suggest a potential role for β_2_-GPI in neovascularization and its therapeutic application for the prevention of angiogenesis-related diseases.

## References

[1] Polz E, Kostner GM. The binding of beta 2-glycoprotein-I to human serum lipoproteins: distribution among density fractions. FEBS Lett. 1979;102: 183–186. 222615 10.1016/0014-5793(79)80955-2

[2] Bouma B, de Groot PG, van den Elsen JM, Ravelli RB, Schouten A, Simmelink MJ, et al Adhesion mechanism of human β_2_-glycoprotein I to phospholipids based on its crystal structure. EMBO J. 1999;18: 5166–5174. 10508150 10.1093/emboj/18.19.5166 PMC1171587

[3] Kato H, Enjyoji K. Amino acid sequence and location of the disulfide bonds in bovine beta 2 glycoprotein I: The presence of five Sushi domains. Biochemistry. 1991;30: 11687–11694. 1751487 10.1021/bi00114a012

[4] Yasuda S, Tsutsumi A, Chiba H, Yanai H, Miyoshi Y, Takeuchi R, et al Beta(2)-glycoprotein I deficiency: prevalence, genetic background and effects on plasma lipoprotein metabolism and hemostasis. Atherosclerosis. 2000;152: 337–346. 10998461 10.1016/s0021-9150(99)00496-7

[5] Takeuchi R, Atsumi T, Ieko M, Takeya H, Yasuda S, Ichikawa K, et al Coagulation and fibrinolytic activities in 2 siblings with beta(2)-glycoprotein I deficiency. Blood. 2000;96: 1594–1595. 10942413

[6] Chiu WC, Chiou TJ, Chiang AN. β_2_-Glycoprotein I inhibits endothelial cell migration through nuclear factor кB signaling pathway and endothelial nitric oxide synthase activation. Biochem J. 2012;445: 125–133. 10.1042/BJ20111383 22489810

[7] Alard JE, Gaillard F, Daridon C, Shoenfeld Y, Jamin C, Youinou P. TLR2 is one of the endothelial receptors for beta2-glycoprotein I. J Immunol. 2010;185: 1550–1557. 10.4049/jimmunol.1000526 20601596

[8] Ma K, Simantov R, Zhang JC, Silverstein R, Hajjar KA, McCrae KR. High affinity binding of beta2-glycoprotein I to human endothelial cells is mediated by annexin II. J Biol Chem. 2000;275: 15541–15548. 10809787 10.1074/jbc.275.20.15541

[9] Eswarappa SM, Fox PL. Antiangiogenic VEGF-Ax: a new participant in tumor angiogenesis. Cancer Res. 2015;75: 2765–2769. 10.1158/0008-5472.CAN-14-3805 26122849 PMC4506224

[10] Zhang Y, Han Q, Ru Y, Bo Q, Wei RH. Anti-VEGF treatment for myopic choroid neovascularization: from molecular characterization to update on clinical application. Drug Des Devel Ther. 2015;9: 3413–3421. 10.2147/DDDT.S87920 26170626 PMC4494177

[11] Chiu WC, Lin JY, Lee TS, You LR, Chiang AN. β_2_-Glycoprotein I inhibits VEGF-induced endothelial cell growth and migration via suppressing phosphorylation of VEGFR2, ERK1/2 and Akt. Mol Cell Biochem. 2013;372: 9–15. 10.1007/s11010-012-1440-6 22956423

[12] Ferrara N, Kerbel RS. Angiogenesis as a therapeutic target. Nature. 2005;438: 967–974. 16355214 10.1038/nature04483

[13] Lamalice L, Le Boeuf F, Huot J. Endothelial cell migration during angiogenesis. Circ Res. 2007;100: 782–794. 17395884 10.1161/01.RES.0000259593.07661.1e

[14] Elshabrawy HA, Chen Z, Volin MV, Ravella S, Virupannavar S, Shahrara S. The pathogenic role of angiogenesis in rheumatoid arthritis. Angiogenesis. 2015;18: 433–448. 10.1007/s10456-015-9477-2 26198292 PMC4879881

[15] Esposito E, Hayakawa K, Maki T, Arai K, Lo EH. Effects of post conditioning on neurogenesis and angiogenesis during the recovery phase after focal cerebral ischemia. Stroke. 2015;46: 2691–2694. 10.1161/STROKEAHA.115.009070 26243221 PMC4604446

[16] Jeong da E, Song HJ, Lim S, Lee SJ, Lim JE, Nam DH, et al Repurposing the anti-malarial drug artesunate as a novel therapeutic agent for metastatic renal cell carcinoma due to its attenuation of tumor growth, metastasis, and angiogenesis. Oncotarget. 2015;6: 33046–33064. 10.18632/oncotarget.5422 26426994 PMC4741748

[17] Brönneke S, Brückner B, Söhle J, Siegner R, Smuda C, Stäb F, et al Genome-wide expression analysis of wounded skin reveals novel genes involved in angiogenesis. Angiogenesis. 2015;18: 361–371. 10.1007/s10456-015-9472-7 26018928

[18] Tong Q, Qing Y, Wu Y, Hu X, Jiang L, Wu X. Dioscin inhibits colon tumor growth and tumor angiogenesis through regulating VEGFR2 and AKT/MAPK signaling pathways. Toxicol Appl Pharmacol. 2014;281: 166–173. 10.1016/j.taap.2014.07.026 25111127

[19] Bekhite MM, Finkensieper A, Binas S, Müller J, Wetzker R, Figulla HR, et al VEGF-mediated PI3K class IA and PKC signaling in cardiomyogenesis and vasculogenesis of mouse embryonic stem cells. J Cell Sci. 2011;124: 1819–1830. 10.1242/jcs.077594 21540297

[20] Kim BR, Seo SH, Park MS, Lee SH, Kwon Y, Rho SB. sMEK1 inhibits endothelial cell proliferation by attenuating VEGFR2-dependent Akt/eNOS/HIF-1α signaling pathways. Oncotarget. 2015;6: 31830–31843. 10.18632/oncotarget.5570 26378810 PMC4741643

[21] Xu J, Yi Y, Li L, Zhang W, Wang J. Osteopontin induces vascular endothelial growth factor expression in articular cartilage through PI3K/AKT and ERK1/2 signaling. Mol Med Rep. 2015;12: 4708–4712. 10.3892/mmr.2015.3975 26099282

[22] Mai J, Qiu Q, Lin YQ, Luo NS, Zhang HF, Wen ZZ, et al Angiotensin II-derived reactive oxygen species promote angiogenesis in human late endothelial progenitor cells through heme oxygenase-1 via ERK1/2 and AKT/PI3K pathways. Inflammation. 2014;37: 858–870. 10.1007/s10753-013-9806-9 24442713

[23] Lin KY, Wang HH, Lai ST, Pan JP, Chiang AN. β_2_-glycoprotein I protects J774A.1 macrophages and human coronary artery smooth muscle cells against apoptosis. J Cell Biochem. 2005;94: 485–496. 15534879 10.1002/jcb.20314

[24] Chistiakov DA, Orekhov AN, Bobryshev YV. Contribution of neovascularization and intraplaque haemorrhage to atherosclerotic plaque progression and instability. Acta Physiol (Oxf). 2015;213: 539–553. 25515699 10.1111/apha.12438

[25] Rivera JC, Noueihed B, Omri S, Barrueco J, Hilberg F, Chemtob S. BIBF1120 (Vargatef) Inhibits preretinal neovascularization and enhances normal vascularization in a model of vasoproliferative retinopathy. Invest Ophthalmol Vis Sci. 2015;56: 7897–7907. 10.1167/iovs.15-17146 26670826

[26] Cao Z, Shang B, Zhang G, Miele L, Sarkar FH, Wang Z, et al Tumor cell-mediated neovascularization and lymphangiogenesis contrive tumor progression and cancer metastasis. Biochim Biophys Acta. 2013;1836: 273–286. 10.1016/j.bbcan.2013.08.001 23933263

[27] Miyakis S, Giannakopoulos B, Krilis S A. β_2_-glycoprotein I-function in health and disease. Thromb Res. 2004;114: 335–346. 15507263 10.1016/j.thromres.2004.07.017

[28] Radziwon-Balicka A, Moncada de la Rosa C, Jurasz P. Platelet-associated angiogenesis regulating factors: a pharmacological perspective. Can J Physiol Pharmacol. 2012;90: 679–688. 10.1139/y2012-036 22512504

[29] Kondo J, Shibata H, Miura S, Yamakawa A, Sato K, Higuchi Y, et al A functional role of the glycosylated N-terminal domain of chondromodulin-I. J Bone Miner Metab. 2011;29: 23–30. 10.1007/s00774-010-0193-0 20506028

[30] Santos IC, Silbiger VN, Higuchi DA, Gomes MA, Barcelos LS, Teixeira MM, et al Angiostatic activity of human plasminogen fragments is highly dependent on glycosylation. Cancer Sci. 2010;101: 453–459. 10.1111/j.1349-7006.2009.01403.x 19961492 PMC11159665

[31] Kassaar O, McMahon SA, Thompson R, Botting CH, Naismith JH, Stewart AJ. Crystal structure of histidine-rich glycoprotein N2 domain reveals redox activity at an interdomain disulfide bridge: implications for angiogenic regulation. Blood. 2014;123: 1948–1955. 10.1182/blood-2013-11-535963 24501222 PMC3962167

[32] Zabrenetzky V, Harris CC, Steeg PS, Roberts DD. Expression of the extracellular matrix molecule thrombospondin inversely correlates with malignant progression in melanoma, lung, and breast carcinoma cell lines. Int J Cancer. 1994;59: 191–195. 7927918 10.1002/ijc.2910590209

[33] Belotti D, Foglieni C, Resovi A, Giavazzi R, Taraboletti G. Targeting angiogenesis with compounds from the extracellular matrix. Int J Biochem Cell Biol. 2011;43: 1674–1685. 10.1016/j.biocel.2011.08.012 21864705

[34] Yu P, Passam FH, Yu DM, Denyer G, Krilis SA. Beta2-glycoprotein I inhibits vascular endothelial growth factor and basic fibroblast growth factor induced angiogenesis through its amino terminal domain. J Thromb Haemost. 2008;6: 1215–1223. 10.1111/j.1538-7836.2008.03000.x 18452581

[35] Hunt JE, Simpson RJ, Krilis SA. Identification of a region of β2-glycoprotein I critical for lipid binding and anti-cardiolipin antibody cofactor activity. Proc Natl Acad Sci (USA). 1993;90: 2141–2145. 8460120 10.1073/pnas.90.6.2141 PMC46041

[36] Wang SX, Cai GP, Sui SF. The insertion of human apolipoprotein H into phospholipid membranes: a monolayer study. Biochem J. 1998;335: 225–232. 9761718 10.1042/bj3350225 PMC1219773

[37] Ma K, Simantov R, Zhang JC, Silverstein R, Hajjar KA, McCrae KR. High affinity binding of beta2-glycoprotein I to human endothelial cells is mediated by annexin II. J Biol Chem. 2000;275: 15541–15548. 10809787 10.1074/jbc.275.20.15541

[38] Itoh Y, Inuzuka K, Kohno I, Wada H, Shiku H, Ohkura N, et al Highly increased plasma concentrations of the nicked form of beta(2) glycoprotein I in patients with leukemia and with lupus anticoagulant: measurement with a monoclonal antibody specific for a nicked form of domain V. J Biochem. 2000;128: 1017–1024. 11098145 10.1093/oxfordjournals.jbchem.a022829

[39] Cai G, Satoh T, Hoshi H. Purification and characterization of an endothelial cell-viability maintaining factor from fetal bovine serum. Biochim Biophys Acta. 1995;1269: 13–18. 7578265 10.1016/0167-4889(95)00091-6

[40] Ioannou Y, Zhang JY, Passam FH, Rahgozar S, Qi JC, Giannakopoulos B, et al Naturally occurring free thiols within beta 2-glycoprotein I in vivo: nitrosylation, redox modification by endothelial cells, and regulation of oxidative stress-induced cell injury. Blood. 2010;116: 1961–1970. 10.1182/blood-2009-04-215335 20551379

[41] Passam FH, Qi JC, Tanaka K, Matthaei KI, Krilis SA. In vivo modulation of angiogenesis by beta 2 glycoprotein I. J Autoimmun. 2010;35: 232–240. 10.1016/j.jaut.2010.06.013 20655705

[42] Gately S, Twardowski P, Stack MS, Cundiff DL, Grella D, Castellino FJ, et al The mechanism of cancer-mediated conversion of plasminogen to the angiogenesis inhibitor angiostatin. Proc Natl Acad Sci (USA). 1997;94: 10868–10872. 9380726 10.1073/pnas.94.20.10868 PMC23512

[43] Sakai T, Balasubramanian K, Maiti S, Halder JB, Schroit AJ. Plasmin-cleaved beta-2-glycoprotein 1 is an inhibitor of angiogenesis. Am J Pathol. 2007;171: 1659–1669. 17872974 10.2353/ajpath.2007.070146 PMC2043526

[44] Beecken WD, Engl T, Ringel EM, Camphausen K, Michaelis M, Jonas D, et al An endogenous inhibitor of angiogenesis derived from a transitional cell carcinoma: clipped beta2-glycoprotein-I. Ann Surg Oncol. 2006;13: 1241–1251. 16955386 10.1245/s10434-006-9009-9

[45] Nakagawa H, Yasuda S, Matsuura E, Kobayashi K, Ieko M, Kataoka H, et al Nicked {beta}2-glycoprotein I binds angiostatin 4.5 (plasminogen kringle 1–5) and attenuates its antiangiogenic property. Blood. 2009;114: 2553–2559. 10.1182/blood-2008-12-190629 19625706

[46] Schuermann A, Helker CS, Herzog W. Metallothionein 2 regulates endothelial cell migration through transcriptional regulation of vegfc expression. Angiogenesis. 2015;18: 463–475. 10.1007/s10456-015-9473-6 26198291 PMC4596909

[47] Tian R, Yang S, Zhu Y, Zou S, Li P, Wang J, et al VEGF/VEGFR2 Signaling Regulates Germ Cell Proliferation in vitro and Promotes Mouse Testicular Regeneration in vivo. Cell Tissue Organ. 2016;201: 1–13. 10.1159/000440949 26727223

[48] Scharpfenecker M, van Dinther M, Liu Z, van Bezooijen RL, Zhao Q, Pukac L, et al BMP-9 signals via ALK1 and inhibits bFGF-induced endothelial cell proliferation and VEGF-stimulated angiogenesis. J Cell Sci. 2007;120: 964–972. 17311849 10.1242/jcs.002949

[49] Delli Carpini J, Karam AK, Montgomery L. Vascular endothelial growth factor and its relationship to the prognosis and treatment of breast, ovarian, and cervical cancer. Angiogenesis. 2010;13: 43–58. 10.1007/s10456-010-9163-3 20229258

[50] Ruan GX, Kazlauskas A. VEGF-A engages at least three tyrosine kinases to activate PI3K/Akt. Cell Cycle. 2012;11: 2047–2048. 10.4161/cc.20535 22647379 PMC3368856

[51] Ding Q, Tian XG, Li Y, Wang QZ, Zhang CQ. Carvedilol may attenuate liver cirrhosis by inhibiting angiogenesis through the VEGF-Src-ERK signaling pathway. World J Gastroenterol. 2015;21: 9566–9576. 10.3748/wjg.v21.i32.9566 26327764 PMC4548117

[52] Iyer AK, Ramesh V, Castro CA, Kaushik V, Kulkarni YM, Wright CA, et al Nitric oxide mediates bleomycin-induced angiogenesis and pulmonary fibrosis via regulation of VEGF. J Cell Biochem. 2015;116: 2484–2493. 10.1002/jcb.25192 25919965 PMC4586046

[53] Beecken WD, Ringel EM, Babica J, Oppermann E, Jonas D, Blaheta RA. Plasmin-clipped beta(2)-glycoprotein-I inhibits endothelial cell growth by down-regulating cyclin A, B and D1 and up-regulating p21 and p27. Cancer Lett. 2010;296: 160–167. 10.1016/j.canlet.2010.04.010 20435405

[54] Lee SJ, Namkoong S, Kim YM, Kim CK, Lee H, Ha KS, et al Fractalkine stimulates angiogenesis by activating the Raf-1/MEK/ERK- and PI3K/Akt/eNOS-dependent signal pathways. Am J Physiol Heart Circ Physiol. 2006;291: H2836–2846. 16877565 10.1152/ajpheart.00113.2006

[55] Chung BH, Kim JD, Kim CK, Kim JH, Won MH, Lee HS, et al Icariin stimulates angiogenesis by activating the MEK/ERK- and PI3K/Akt/eNOS-dependent signal pathways in human endothelial cells. Biochem Biophys Res Commun. 2008;376: 404–408. 10.1016/j.bbrc.2008.09.001 18789310

[56] Somanath PR, Razorenova OV, Chen J, Byzova TV. Akt1 in endothelial cell and angiogenesis. Cell Cycle. 2006;5: 512–518. 16552185 10.4161/cc.5.5.2538 PMC1569947

[57] Murohara T, Horowitz JR, Silver M, Tsurumi Y, Chen D, Sullivan A, et al Vascular endothelial growth factor/vascular permeability factor enhances vascular permeability via nitric oxide and prostacyclin. Circulation. 1998;97: 99–107. 9443437 10.1161/01.cir.97.1.99

[58] Noiri E, Lee E, Testa J, Quigley J, Colflesh D, Keese CR, et al Podokinesis in endothelial cell migration: role of nitric oxide. Am J Physiol. 1998;274: C236–244. 9458733 10.1152/ajpcell.1998.274.1.C236

[59] Morbidelli L, Chang CH, Douglas JG, Granger HJ, Ledda F, Ziche M. Nitric oxide mediates mitogenic effect of VEGF on coronary venular endothelium. Am J Physiol. 1996;270: H411–415. 8769777 10.1152/ajpheart.1996.270.1.H411

[60] Bir SC, Xiong Y, Kevil CG, Luo J. Emerging role of PKA/eNOS pathway in therapeutic angiogenesis for ischaemic tissue diseases. Cardiovasc Res. 2012;95: 7–18. 10.1093/cvr/cvs143 22492672 PMC3378310

[61] Huang D, Ding Y, Luo WM, Bender S, Qian CN, Kort E, et al Inhibition of MAPK kinase signaling pathways suppressed renal cell carcinoma growth and angiogenesis in vivo. Cancer Res. 2008;68: 81–88. 10.1158/0008-5472.CAN-07-5311 18172299

[62] Wang CY, Tsai AC, Peng CY, Chang YL, Lee KH, Teng CM, et al Dehydrocostuslactone suppresses angiogenesis in vitro and in vivo through inhibition of Akt/GSK-3beta and mTOR signaling pathways. PLoS One 2012;7: e31195 10.1371/journal.pone.0031195 22359572 PMC3281050

